# Investigation of Immunomodulatory and Gut Microbiota-Altering Properties of Multicomponent Nutraceutical Prepared from Lactic Acid Bacteria, Bovine Colostrum, Apple Production By-Products and Essential Oils

**DOI:** 10.3390/foods10061313

**Published:** 2021-06-07

**Authors:** Juozas Grigas, Modestas Ruzauskas, Arnoldas Pautienius, Elena Bartkiene, Vita Lele, Vytaute Starkute, Paulina Zavistanaviciute, Egle Zokaityte, Jurga Bernatoniene, Liudas Ivanauskas, Valdas Jakstas, Arunas Stankevicius

**Affiliations:** 1 Laboratory of Immunology, Department of Anatomy and Physiology, Lithuanian University of Health Sciences, Tilzes Str. 18, LT-47181 Kaunas, Lithuania; juozas.grigas@lsmuni.lt (J.G.); modestas.ruzauskas@lsmuni.lt (M.R.); arnoldas.pautienius@lsmuni.lt (A.P.); 2 Institute of Microbiology and Virology, Lithuanian University of Health Sciences, Tilzes Str. 18, LT-47181 Kaunas, Lithuania; 3 Department of Food Safety and Quality, Lithuanian University of Health Sciences, Tilzes Str. 18, LT-47181 Kaunas, Lithuania; elena.bartkiene@lsmuni.lt (E.B.); vita.lele@lsmuni.lt (V.L.); vytaute.starkute@lsmuni.lt (V.S.); paulina.zavistanaviciute@lsmuni.lt (P.Z.); egle.zokaityte@lsmuni.lt (E.Z.); 4 Institute of Animal Rearing Technologies, Lithuanian University of Health Sciences, Tilzes Str. 18, LT-47181 Kaunas, Lithuania; 5 Institute of Pharmaceutical Technologies, Lithuanian University of Health Sciences, Sukilėlių Pr. 13, LT-50162 Kaunas, Lithuania; jurga.bernatoniene@lsmuni.lt (J.B.); valdas.jakstas@lsmuni.lt (V.J.); 6 Department of Drug Technology and Social Pharmacy, Institute of Pharmaceutical Technologies, Lithuanian University of Health Sciences, Sukilėlių Pr. 13, LT-50162 Kaunas, Lithuania; 7 Department of Analytical and Toxicological Chemistry, Lithuanian University of Health Sciences, Sukilėlių Pr. 13, LT-50162 Kaunas, Lithuania; liudas.ivanauskas@lsmuni.lt; 8 Department of Pharmacognosy, Lithuanian University of Health Sciences, Sukilėlių Pr. 13, LT-50162 Kaunas, Lithuania

**Keywords:** nutraceutical, lactic acid bacteria, prebiotic, bovine colostrum, apple, essential oil, microbiota, immunomodulation

## Abstract

Dietary components, such as lactic acid bacteria (LAB), bovine colostrum, apple production by-products, and essential oils, can favorably alter the host immune system and gut microbiota, however, their cumulative effect as multicomponent nutraceutical supplement has not been investigated. Therefore, the present study is the first one to evaluate a combination of LAB, bovine colostrum, dehydrated apple pomace, and essential oils for their immunomodulatory and prebiotic properties in the swine model. This study shows that supplementary feeding of pigs using multicomponent nutraceutical resulted in a statistically significant decrease in proportions of T cytotoxic and double-positive (CD4^+^CD8^+low^) cells within the CD3^+^ cell population at 28 DPI, compared to the beginning of the experiment (0DPI). Conversely, a statistically significant increase in proportions of B cells (accompanied by an increase in IgG concentration) and macrophage/monocyte cells within viable cell population at 28 DPI, compared to the beginning of the experiments, was observed. Furthermore, changes in the bacterial composition of gut microbiota in pigs fed with multicomponent nutraceutical changed significantly, with a 1.78 times higher number of probiotic strains (*Bifidobacterium*, *Streptococcus, Faecilbacterium*) at the end of the experiment, compared to control group animals. This study shows a positive effect of the nutraceutical formula used on the changes of gut microbiota by facilitating an increase in probiotic bacteria strains and possible anti-inflammatory properties.

## 1. Introduction

Nutraceuticals are dietary supplements containing concentrated bioactive compounds found in a variety of foods and presented in a pharmaceutical form, allowing the recipient of a nutraceutical to receive dosages of bioactive compounds that would normally not be possible when consuming regular quantities of food [1]. Nutraceuticals are used for health benefits they provide including, prevention, treatment of diseases, and immunomodulatory properties. A variety of different dietary supplements has also been found to have a significant effect on gut [2] and airway [3] microbiota of tested individuals, associated with resulting health benefits.

Probiotics are defined as living microorganisms that, when administered, provide the host with health benefits [4]. Despite a wide variety of mechanisms of action, some probiotic strains have been shown to provide a favorable environment for gut microbiota, in turn ameliorating symptoms of infectious diarrhea, antibiotic-associated diarrhea, irritable bowel syndrome (IBS), and other conditions of the digestive tract [5]. In addition, immunomodulatory effects of probiotics have been demonstrated, with a resulting impact on a diverse group of conditions, including atopic dermatitis, inflammatory bowel disease, rheumatoid arthritis, and autoimmune encephalomyelitis [6,7,8]. In particular, probiotic strains belonging to genus *Lactobacillus* have been widely investigated for their immunomodulatory properties, including suppression of T helper 2 (Th2) cell-produced cytokines (IL-4 and IL-5), increase in CD4^+^ and CD8^+^ cell counts and levels of IL-12 and INF-γ, and improvements to phagocytic activity [9,10,11,12]. *L. casei* has been found to increase the number of CD4, SWC3, CD11R1, and MHCII expressing mononuclear and dendritic cells in both intestinal tissue and the blood of pigs [13]. Probiotic-dependent alterations of microbiota have not yet been extensively studied. To date, the effects of various *Lactobacillus* strains on the stability of fecal microbiota have been investigated, indicating the positive effect of probiotic strains on the index of evenness. In particular, *L. casei* subsp. *rhamnosus*, when used as a probiotic, has been associated with the elevated abundance of Lactobacillaceae and Bifidobacteriaceae species known to be beneficial in ameliorating gastrointestinal atopy, IBS, and other gastrointestinal disorders [14].

Bovine colostrum as a supplement has been known to have adipogenic, antioxidant, and anti-inflammatory properties, associated with a wide variety of biologically active molecules found in its composition [15]. In particular, immunoglobulins, lactoferrin, lysozyme, antioxidants, oligopolysaccharides, proline-rich peptides, and cytokines have been associated with antimicrobial and immunomodulatory effects [16]. Bovine colostrum is rich in immunoglobulins, particularly immunoglobulin G (IgG), which acts as a protective agent for a newborn calf when colostrum is ingested in sufficient quantities. Recently, hyperimmune bovine colostrum, resulting from stimulation of colostrum-producing cow with the desired antigen in order to produce high levels of colostral immunoglobulins against the target antigen, has been generated and used as a preventative agent against pathogenic species of *Clostridium*, *E. coli*, and *Shigella* [17]. Lactoferrin, another biologically active agent found in colostrum, is responsible for a wide variety of immunomodulatory properties, including suppression of proinflammatory cytokines and type I interferons, promotion of morphological changes of dendritic cells, suppressed production of T cell-produced cytokines, and enhanced B cell antigen presentation [18]. In addition, bovine colostrum contains prebiotic compounds, such as fructo-oligosaccharides, galacto-oligosaccharides, gangliosides, and nucleosides that promote the growth of gut microbiota, particularly varieties of Bifidobacteriacea and Lactobacillaceae family [16].

The variety of phytochemicals in vegetables and fruit, particularly phenols and carotenoids, has been associated with a range of health benefits. Apple procyanidins, in particular, have been associated with protective properties against gastrointestinal disorders, such as inflammatory bowel disease and food allergy-associated sensitization [19,20]. Oral administrations of apple procyanidins ameliorate inflammatory bowel disease symptoms via different immunomodulatory pathways: by suppressing the production of intestinal epithelial cell-produced IL-8, in turn suppressing neutrophil recruitment into intestinal tissue, and by reducing the production of INF-γ by CD4^+^ and CD8^+^ cells [21]. Other phenols found in apples, such as condensed tannins, have also been associated with immunomodulatory properties, such as suppression of T helper 17 cells, in turn delaying the development of rheumatoid arthritis [22]. The positive effect of phenols and other biologically active compounds found in apples have also been associated with changes in host microbiota and has been demonstrated in both in vitro [23] and in vivo studies [24,25].

Essential oils have been reported for their beneficial biological functions including, antimicrobial, antiviral, anti-inflammatory, and antioxidant effects [26]. Most essential oils contain chemical compounds that belong to terpenoids and phenylpropanoids. Oregano is a rich source of active compounds such as polyphenols (flavonoids and phenolic acids), essential oil (with carvacrol and/or thymol, linalool, and p-cymene), triperpenoids, and sterols [27]. This herb has been suggested to have various beneficial properties for health ranging from, anti-inflammatory, antioxidant, antispasmodic, to anti-urolithic, analgesic, and antimicrobial effects [27,28]. Mint essential oil consisted of high amounts of menthol, menthone, 1,8-cineole, carvone, and other compounds [26,29]. Mint essential oil was tested to be an effective treatment of IBS in humans. This effect is considered to be associated with the antioxidant and anti-inflammatory activities of mint essential oil [26]. Thymus vulgaris herb is a rich source of essential oil. The main compounds of Thymus essential oil are carvacrol, thymol, geraniol, linalool, and gamma-terpineol [30,31]. The main components of Thyme essential oil are responsible for antimicrobial, antiviral, antioxidant, and anti-inflammatory properties [31,32]. Thyme essential oil shows beneficial effects for gastric and intestinal environments [33]. Thyme essential oil, which predominant compounds are thymol and carvacrol, increase the activity of pancreatic trypsin, lipase and protease, show spasmolytic effects, reduce intestinal infections of gram-positive and gram-negative bacteria, fungi and yeasts, hookworms, ascarids, and decrease the edema in various types of inflammation [33]. Thyme essential oil could be used for curing ulcerative colitis and Crohn’s disease [33].

In the present study, we test the effect of a nutraceutical prepared from a combination of 4 components in vivo by periodic oral administrations to pigs as a feed supplement and evaluate its immunomodulatory and prebiotic properties by comparing changes in immune cell phenotype populations and diversity of fecal microbiota between tested and negative control animal groups. To date, the combination of components tested in the present study has not been evaluated in vitro or in vivo. Moreover, some of the components and similar active substances have been tested separately in mice and rat models, however, only limited data is available from trials using swine as in vivo models. Therefore, the present study is the first one to evaluate a combination of lactic acid bacteria (LAB), bovine colostrum, dehydrated apple pomace, and essential oils for their immunomodulatory and prebiotic properties in the swine model.

## 2. Materials and Methods

### 2.1. Nutraceutical Composition

Nutraceutical used in this study consists of 4 components: (A) LAB strains (*L.uvarum* LUHS245 and *L.casei* LUHS210), multiplied in whey and lyophilized as previously described [34]. The average viable LAB count in a lyophilized powder form was 8.89 log10 cfu/g. LAB strains used were selected based on their antimicrobial properties as previously described [35]. Briefly, LAB antimicrobial properties were measured based on the diameters of pathogenic and opportunistic bacteria inhibition zones generated by tested LAB strains. Inhibition zone diameter of >10 mm against all tested bacteria was chosen as a qualifying criterion for LAB strain viability as a nutraceutical component. (B) Bovine colostrum fermented using LUH245 strains and prepared as previously described [34]. (C) Dehydrated apple pomace (DAP) selected based on their antimicrobial properties against pathogenic and opportunistic strains as previously described [36]. Briefly, berry and fruit by-product antimicrobial properties were measured based on the diameter of pathogenic and opportunistic bacteria inhibition zones they generated [36]. DAP was, in turn, chosen as the most viable candidate as a nutraceutical component. (D) Oregano extract (containing 131.84 mg/mL carvacrol), mint essential oil (containing 10.22 mg/mL menthol) and thyme essential oil (containing 404.0 mg/mL thymol and 9.73 mg/mL carvacrol) (quantity ratios 98.95: 0.312: 0.738) in a tablet form. Tablets were produced using the single punch eccentric tablet press Erweka RTP-D8 (Langen, Germany) (the press was speed 4 units, the compression force was 5 N) [37]. Daily supplement preparations were prepared from 10 g of components A, B, and C and a single tablet of component D.

### 2.2. In Vivo Experiment Design

16 6-week-old white breed pigs with an initial weight of 18.1 ± 1.6 kg were kept under specific pathogen-free conditions (SPF) in the animal facility at the Center of Animal Research of Lithuanian University of Health Sciences. Animals were separated into four groups of four pigs and housed in separate stalls. Groups 1 and 2 consisted of female and male controls, respectively, while groups 3 and 4 consisted of female and male test animals, respectively. All animals were fed conventional concentrated feed, while groups 3 and 4 were fed daily 30 g of supplement preparation with a mixed in essential oil tablet in addition to the regular feed for the duration of the experiment. The experiment was terminated after four weeks; all animals were euthanized and necropsied. All experimental procedures were carried out according to the requirements of animal welfare (National Food and Veterinary Risk Assessment Institute of Lithuania Permission Issue Number G2-123).

### 2.3. Sample Collection

Whole blood and fecal samples were collected prior to specialized feeding and on days 7 (fecal sample only), 14, and 28 after experiment initiation. All collected blood samples were used for IgA, IgG, and IgM concentration measurements and flow cytometric analysis, while fecal samples were used for microbiome analysis.

### 2.4. Cholesterol, Immunoglobulin and Weight Gain Measurements

Cholesterol and immunoglobulin measurements were carried out using AU-680 Clinical Chemistry Analyzer (Backman Coulter, Brea, CA, USA) at 28 DPI. For IgG, IgA, and IgM measurements, goat anti-IgG, goat anti-IgA, and goat anti-IgM antisera were used, respectively, according to the manufacturer’s instructions. Total and high-density lipoprotein (HDL) cholesterol measurements were carried out using the cholesterol esterase protocol, according to the manufacturer’s instructions. Weight measurements were carried out at 7, 14, and 28 DPI.

### 2.5. Peripheral Blood Mononuclear Cell Isolation, Cell Staining and Flow Cytometry

Peripheral blood mononuclear cells (PBMCs) were isolated from whole blood samples using Ficoll-Paque PLUS (GE Healthcare, Chicago, IL, USA) using density gradient centrifugation. PBMCs were suspended in phosphate buffer (PBS, 1×, pH 7.2; Gibco, Grand Island, NY, USA) and concentrations of 4 × 106 cells/mL were determined. PBMCs were used for staining immediately after isolation. Cell staining was performed using fluorochrome-conjugated antibodies: fluorescein isothiocyanate (FITC) labeled anti-porcine CD4, allophycocyanin (APC) labeled anti-porcine CD8α, phycoerythrin (PE) labeled anti-porcine CD3, PE-Cyanine7 labeled anti-porcine CD2, FITC labeled anti-porcine CD14, and AlexaFluor 674 labeled anti-porcine CD21. Stained cells were analyzed using a flow cytometer (FACS Calibur, BD Biosciences, Franklin Lakes, NJ, USA) by acquiring 10,000 events for each experiment and analyzing data using FlowJo v10 (Tree Star) software.

**Figure 1:**
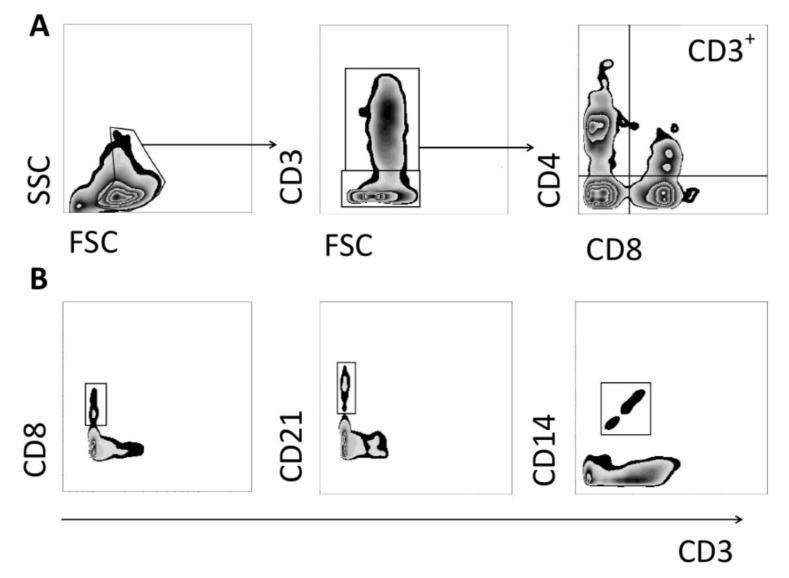
Gating strategy applied to flow cytometry data. (**A**) Viable cells were selected based on FSC and SSC, followed by establishing a gate of CD3^+^ and CD3^−^ gates. Further gates based on CD4 and CD8 markers were established within CD3^+^ gate. (**B**) Gates based on CD3 and CD8, CD21, CD14 markers were established within viable cell gate.

CD3^+^ and CD3^−^ cell gates were established within the viable cell population. CD4^−^ CD8^+^ (phenotype that mostly contains T cytotoxic cells), CD4^+^ CD8^+low^ (phenotype that mostly contains memory T cells), and CD4^+^ CD8^−^ (phenotype that mostly contains T helper cells) gates were established within the CD3^+^ cell population. All T cell population sizes were expressed as proportions of CD3+ cells and total cells. CD3^−^CD21^+^ (phenotype that mostly contains B cells) and CD3^−^CD4^−^ CD8^+low^ (phenotype that mostly contains NK cells) gates were established within the viable cell population and expressed as a proportions of the total cells. CD14^+^ (phenotype that mostly contains macrophage/monocyte cells) gate was established within the viable cell population and expressed as proportion of total cell population. The gating strategy used in this study is summarized in Figure 1. CD8 was designated as either CD8^+^ or CD8^+low^ based on fluorescence intensity of >102 or <102, respectively.

### 2.6. Metagenomics and Microbial Profiling Analysis

DNA from each fecal sample was purified using a fecal DNA MiniPrep kit (D6010, Zymo Research, Irvine, CA, USA) according to the manufacturer‘s instructions. Obtained DNA was then purified using DNA Clean & Concentrator-25 kit (Zymo Research, Irvine, CA, USA) to produce DNA concentrations of at least 50 ng/μL. Metagenomic libraries were prepared, sequenced, and assembled in an independent service laboratory (Baseclear, Leiden, The Netherlands). Short paired sequence reads were generated using the MiSeq system (Illumina, San Diego, CA, USA) and converted to FASTQ files using BCL2FASTQ pipeline software (Illumina, San Diego, CA, USA). Quality trimming was applied based on Phred quality scores. Subsequently, the Illumina paired reads were merged into single reads (so-called ‘pseudoreads’) through sequence overlap. Chimeric pseudoreads were removed, and the remaining reads were aligned to the combination of GreenGenes and RDP 16S gene databases. Based on the alignment scores of pseudoreads, the taxonomic classes were assigned by associating each pseudoread to the best matching Operational Taxonomic Unit (OTU).

### 2.7. Statistical Analysis

Paired comparisons of flow cytometric data were performed using a two-tailed Wilcoxon rank-sum test. Differences between metagenomic data groups were assessed using the Z-Test Calculator for 2 population proportions (Social Science Statistics, 2020). *p* values < 0.05 were considered to be statistically significant. Standard deviations were calculated for all descriptive analyses.

## 3. Results

### 3.1. Changes in CD3^+^ T Cell Population Phenotypes

**Figure 2:**
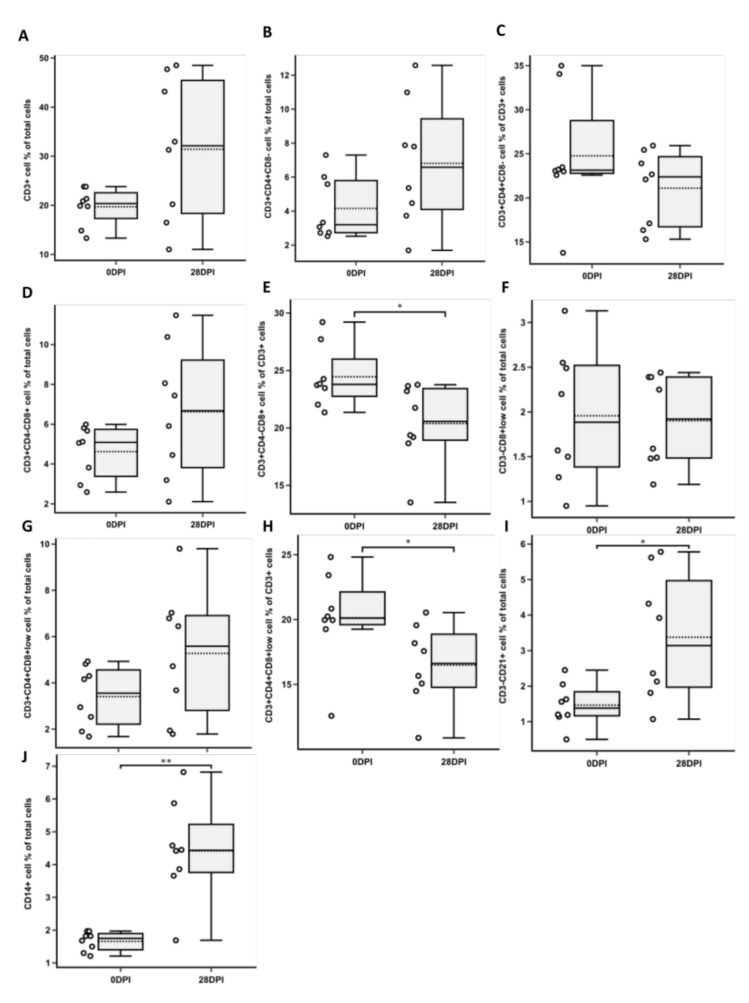
Immunophenotyping of cells obtained from pigs before (0DPI) and 28 days after (28DPI) supplementary feeding by flow cytometry. Box plots show the percentage (mean ± SD) of CD3^+^ cells (**A**), T helper cell (CD3^+^CD4^+^CD8^−^) proportion of total (**B**) and CD3+ (**C**) cells, T cytotoxic cell (CD3^+^CD4^−^CD8^+^) proportion of total (**D**) and CD3^+^ (**E**) cells, percentage of NK cells (CD3^−^CD4^−^CD8^+low^) (**F**), T memory cell (CD3^+^CD4^+^CD8^+low^) proportion of total (**G**) and CD3^+^ (**H**) cells, percentage of B cells (CD3^−^CD21^+^) (**I**), and percentage of macrophage/monocyte cells (CD14^+^) (**J**). Asterisks indicate statistical significance between groups based on two-tailed Wilcoxon rank sum test results (* *p* < 0.05; ** *p* < 0.005).

**Figure 3:**
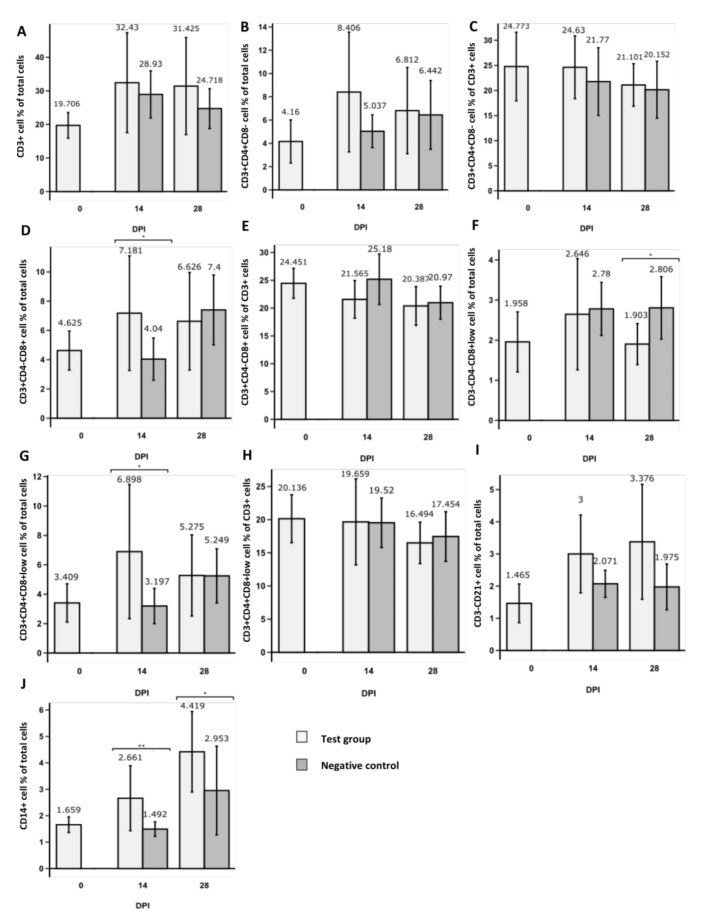
Comparison between immune cell subpopulations of test animal and negative control groups obtained from pigs before (0DPI), 14 days (14DPI) and 28 days (28DPI) after supplementary feeding by flow cytometry. Bar plots show the percentage (mean ± SD) of CD3^+^ cells (**A**), T helper cell (CD3^+^CD4^+^CD8^−^) proportion of total (**B**) and CD3^+^ (**C**) cells, T cytotoxic cell (CD3^+^CD4^−^CD8^+^) proportion of total (**D**) and CD3^+^ (**E**) cells, percentage of NK cells (CD3^−^CD4^−^CD8^+low^) (**F**), T memory cell (CD3^+^CD4^+^CD8^+low^) proportion of total (**G**) and CD3^+^ (**H**) cells, percentage of B cells (CD3^−^CD21^+^) (**I**), and percentage of macrophage/monocyte cells (CD14^+^) (**J**). Asterisk indicates statistical significance between groups based on two-tailed Wilcoxon rank sum test results (* *p* < 0.05; ** *p* < 0.01).

Mean values of CD3^+^ cell frequency were comparable between test and negative control groups and between 0 DPI (days post-initiation) and 28 DPI groups (Figure 2). No statistically significant differences in frequencies of CD3^+^CD4^+^CD8^−^ cells within total tested cells or CD3^+^ cells were observed between 0 DPI and 28 DPI groups (Figure 2B,C). No statistically significant differences in frequencies of CD3^+^CD4^−^CD8^+^ cells within total tested cells were observed between 0 DPI and 28 DPI groups (Figure 2D). However, a significant (*p* < 0.05) decrease in CD3^+^CD4^−^CD8^+^ cells was observed within CD3^+^ cells between 0 DPI and 28 DPI groups (Figure 2E). Similarly, a statistically significant (*p* < 0.05) decrease in CD3^+^CD4^+^CD8^+low^ cells was observed within CD3^+^ cells between 0 DPI and 28 DPI groups (Figure 2H), but not within total tested cells (Figure 2G). Interestingly, a statistically significant increase (*p* < 0.05) was observed between tested and negative control group CD3^+^CD4^−^CD8^+^ cell frequencies within total cells 14 DPI (Figure 3D), but not within total cells 28 DPI and within CD3^+^ cell population (Figure 3D,E). Similarly, a statistically significant increase (*p* < 0.05) was observed between tested and negative control group CD3^+^CD4^+^CD8^+low^ cell frequencies within total cells 14 DPI (Figure 3G), but not within total cells 28 DPI and within CD3^+^ cell population (Figure 3G,H).

### 3.2. Changes in NK and B Cell Populations

NK cell and B cell populations were gated within viable cell gates. No statistical difference in mean frequency values of CD3^−^CD4^−^CD8^+low^ cells was observed between 0 DPI and 28 DPI groups (Figure 2F). However, a statistically significant decrease (*p* < 0.05) of the aforementioned cells between tested and negative control groups was observed at 28 DPI, but not at 14 DPI (Figure 3F). A statistically significant increase (*p* < 0.05) in CD3^−^CD21^+^ cells was observed between 0 DPI and 28 DPI groups (Figure 2I). However, no significant difference between tested and negative control groups was observed at both 14 DPI and 28 DPI (Figure 3I).

### 3.3. Changes in CD14^+^ Population

CD14^+^ population was gated within the total viable cell group. A statistically significant increase (*p* < 0.005) in CD14^+^ cells was observed between 0 DPI and 28 DPI groups within total tested cells (Figure 2J). Moreover, a statistically significant increase in CD14^+^ cells between tested and negative control groups was observed both at 14 DPI (*p* < 0.01) and at 28 DPI (*p* < 0.05) (Figure 3J).

### 3.4. Changes in Immunoglobulin, Cholesterol and Weight Gain Measurements

Statistically significant changes in weight gain between control and test groups at 28 DPI were not observed (Table 1). An increase in total cholesterol level (2.34 ± 0.34 mmol/L) was observed in the test group compared to the control group (1.89 ± 0.38 mmol/L). However, statistically significant changes of HDL cholesterol were not observed between test and control groups 28 DPI (Table 1). An increase in all immunoglobulin subpopulations was observed in test groups compared to control groups 28 DPI. However, only an increase of IgG levels was statistically significant (Table 1).

### 3.5. Microbial Profiles

**Figure 4:**
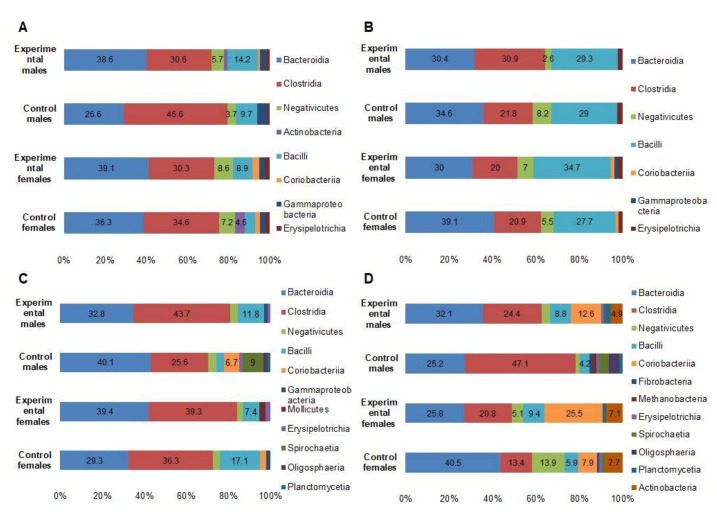
Bacterial classes in test animal and negative control group intestinal microbiota before (**A**), 7 days (**B**), 14 days (**C**) and 28 days (**D**) after supplementary feeding.

*Bacteroidia* and *Clostridia* were the two most prevalent bacterial classes in feces samples on 0 DPI, with prevalence rates ranging from 69.2% to 73.2% in all animal groups, followed by *Negativicutes*, *Bacilli*, *Gammaproteobacteria*, and *Erysipelotrichia*, with prevalence rates ranging from 15.9% to 24.5% (Figure 4A). Overall, the number of identified bacteria on 0 DPI ranged from 22,205 to 28,266 OTU, with an average of 25,383.25 (±2775.306) OUT. *Prevotella* was the most predominant genus within the aforementioned classes in all animal groups (16.1% to 27.9%), followed by *Clostridia* (7.59% to 12.47%), *Barnesiella* (2.4% to 10.3%), and *Streptococcus* (2.24% to 8.76%).

**Figure 5:**
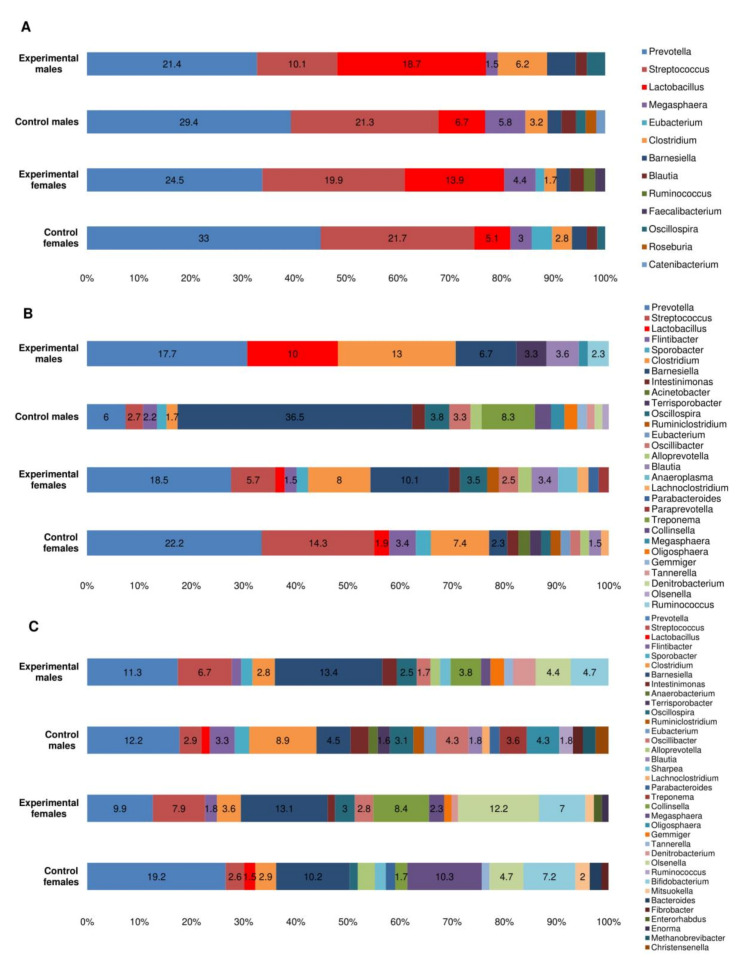
Bacterial genera in test animal and negative control group intestinal microbiota 7 days (**A**), 14 days (**B**) and 28 days (**C**) after supplementary feeding. Genera with prevalence rate of >1% are presented.

At 7 DPI, a minor increase in the number *Bacilli* was detected, with no obvious differences at a class level among all animal groups (Figure 4B). Changes at a genus level were more pronounced, particularly for *Lactobacillus*, with a prevalence rate 2.8 times higher (*p* < 0.05) in test groups compared to negative controls (Figure 5A). Species variety among *Lactobacillus* was also higher in test groups compared to negative controls, with 19 and 15 species observed, respectively. *Prevotella* and *Streptococcus* remained the most prevalent genera among both test and control groups, with slightly lower rates in test groups compared to controls (*p* < 0.05). In addition, a larger bacterial variety at a genus level was observed in the test male group, compared to other groups.

*Bacteroidia* and *Clostridia* remain the most prevalent bacterial classes in all animal groups 14 DPI, with statistically higher (*p* < 0.05) prevalence rates of *Clostridia* in test groups compared to negative controls (Figure 4C). Interestingly, the male control group expressed a different microbial profile, with the presence of *Spirochaetia*, *Oligosphaeria*, and *Plantomycetia*, which was not observed in other animal groups (Figure 4C). In addition, on the genus level, the male control group expressed predominance of *Bernesiella* (with the presence of a single species *B. intestinihominis*) with a prevalence rate of 36.5% compared to the prevalence rates ranging from 2.3% to 10.1% in other groups (*p* < 0.05) (Figure 5B). In addition, *Treponema* (*T. succinifaciens*) was only detected in the male control group (prevalence rate of 8.3%). Elevated *Lactobacillus* prevalence rate (10%) was significantly (*p* < 0.05) higher in the male test group, but not in control groups and in the female test group (Figure 5B).

The number of identified bacteria on 28 DPI in all animal groups ranged from 21,657 to 26,875 OTU. The number of identified genera was comparable between the test and control groups, ranging from 244 to 264. There were no statistically significant differences between the test and control groups with respect to predominant bacterial class distributions (Figure 4D). However, some classes (*Fibrobacteria*, *Spirochaetia*) with prevalence rates of >1% were only present in control groups and not in test groups (see Supplementary Material Tables S1 and S2). The most prevalent genera in test groups were *Barnesiella*, *Olsenella*, *Prevotella*, *Streptococcus,* and *Collinsella*, while the most prevalent genera in control groups were *Prevotella*, *Clostridium*, *Megasphaera*, and *Barnesiella* (Figure 5C).

**Figure 6:**
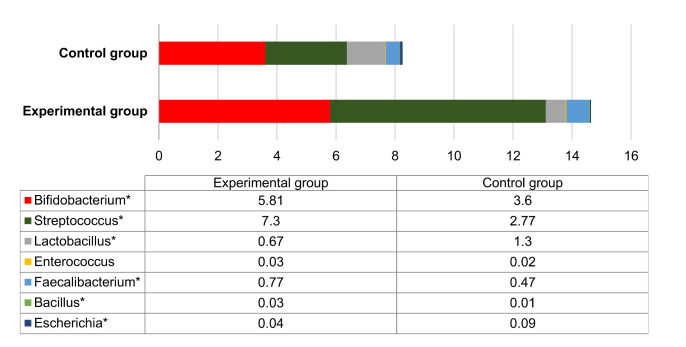
The percentage of probiotic bacteria in negative control and test animal groups at the end of experiment (28 DPI). Asterisk indicates statistical significance between groups (* *p* < 0.05).

Generally, the number of bacteria that are considered to be probiotic was 1.78 times higher in test groups compared to negative controls (*p* < 0.05) (Figure 6). The prevalence of *Escherichia* and *Lactobacillus* was higher (*p* < 0.05) in fecal samples of control group animals compared to test groups (Figure 6).

## 4. Discussion

In this study, we used two criteria for evaluating the effect of multi-component nutraceutical in vivo. The immunomodulatory effect was evaluated by comparing changes in immune cell phenotype populations using CD3, CD4, CD8, CD14, and CD21 markers, while probiotic properties were determined by comparing the diversity of fecal microbiota on family, genera, and species level.

Minor changes in cells of adaptive immunity were observed in the test group of pigs that received oral administration of nutraceutical preparation. A statistically significant decrease was observed in CD3^+^CD4^−^CD8^+^ and CD3^+^CD4^+^CD8^+low^ within CD3^+^ population 28 DPI, indicating relative suppression of T cytotoxic and T memory cell production by oral feed supplementation. The effects of probiotic Lactobacillus strains on host immune systems have previously been investigated, with some data indicating an immunomodulatory effect of different species, including reduction of levels of pro-inflammatory cytokines (TNF-α and IL-17) and Th2 cell produced cytokines (IL-4 and IL-5), increase in cytokine IL-10 and IL-12 production, increase in CD4^+^ and CD8^+^ cell count, and improvements to phagocytotic activity [7,9,10,11,12]. Contrary to our findings, the study that used *L. casei* for the amelioration of colon carcinoma symptoms showed an increased proportion of CD8^+^ cells in probiotic-fed mice, indicating an increase in recruitment of T cytotoxic and other CD8^+^ cells [38]. However, the effect of *L. uvarum* as a supplement on the immune system of the host has not yet been investigated. Therefore, the effect of *L. casei* and *L. uvarum* on the recruitment of CD8^+^ cell population might act in an antagonistic manner, requiring a separate investigation on the immunomodulatory characteristics of *L. uvarum*.

Most studies of bovine colostrum as an immunomodulatory substance focused on the effect of colostral antibodies as a protective agent against infections of neonates. Bovine colostrum as a supplement is mostly used in a form of hyperimmune colostrum, generated against a specific antigen. However, there is a lack of data on the effects of colostrum in an unaltered form on adult individuals and the capacity of colostrum to induce an immunomodulatory effect in cross-species transfer. Studies testing the effects of different components of bovine colostrum on cytokine production of human PBMCs revealed opposing results depending on the component used, indicating multifaceted possible immunomodulatory effects [39]. Bovine colostrum protein concentrate has been associated with an increase in cytokines associated with cell-mediated immunity, mostly IFN-γ and IL-2, which are associated with Th1 cell-produced cytokines. However, in our study, no statistically significant change in the number T helper cell population has been detected. Despite the lack of quantitative changes in T regulatory cell population and direct measurements of IFN-γ concentration, an increase in IL-10 production could be assumed based on the reduced number of CD3^+^CD4-CD8^+^ cell population, which correlates with reduced IFN-γ levels [40].

Contrary to our findings, data acquired from studies using orally administered apple procyanidins demonstrate an increase in the proportions of γδTCR+ and αβTCR+ T cells in the intraepithelial lymphocyte population of mice [21]. Intraepithelial lymphocytes are located in the intestinal mucosa, while the data in the present study was acquired by using PBMCs, complicating the direct comparison between these data. However, assumptions could be made that decrease in CD3^+^CD4^−^CD8^+^ and CD3^+^CD4^+^CD8^+low^ cells, which are part of the porcine αβTCR+ T cell group [41], could be associated with recruitment of cells to the intestinal mucosa, resulting in an increase of αβTCR+ T cell proportion in intestinal mucosa and a decrease in PBMC population. Another limitation lies in species difference, making a comparison between the effect of apple extract on porcine and murine models complicated. In addition, apple extract in this study consists of other active compounds found in apples, possibly acting in an antagonistic manner towards procyanidins.

In general, nutraceutical preparation used in this study appears to suppress the T cell proportion in the PBMC population rather than increase it, as shown by different studies that utilized similar components to those used in this study. However, the majority of supplementation testing in the aforementioned in vivo studies was carried out using mice as an animal model, indicating possible differences in the effect of nutraceutical components on murine and porcine T cells.

Contrary to the decrease in T cell subpopulations, an increase in CD3^−^CD21^+^ cells, which correspond to the B cell group, was observed within CD3^−^population 28 DPI. Similarly, a statistically significant increase in IgG concentration was observed in the test group, indicating a general increase in the humoral immune response. Quantitative changes in B cells among CD3^−^ population could be attributed to specific responses against microbial epitopes of lactobacilli. In particular, *L.uvarum*—bacterium isolated from Spanish Bobal grape must—could be responsible for host humoral immune response, since no data currently describes its availability in natural host microbiota [42]. In addition, high antibody concentration in bovine colostrum could also trigger the humoral immune response in a porcine organism. Fc domain of bovine IgG is a primary recognition site, triggering highly selective secondary antibody production, as bovine IgGs are treated as antigens by the antigen-presenting cells of the porcine host [43]. Therefore, an elevated level of B cells and IgG concentration is to be expected, considering the antigenic properties of foreign antibodies. The relative decrease in CD3^+^CD4^−^CD8^+^ and CD3^+^CD4^+^CD8^+low^ within the CD3^+^ population could also be attributed to the relative increase in CD3^−^CD21^+^ cells, indicating a shift towards B cell proliferation in the peripheral bloodstream at the cost of T cell number.

The most significant difference between 0 DPI and 28 DPI groups was observed among the CD14^+^ population, corresponding to macrophage/monocyte cells. Opposing effects on phagocytic activity have previously been reported based on the single components that were used in combination as in the current study. To date, probiotic *L. acidophilus*, *L. reuteri*, *L. rhamnosus*, and *Bifidobacterium lactis* have previously been associated with an increase in antigen-presenting cell proportions in pig lymphoid tissue [13,44]. Our findings coincide with groups of pigs used in Zhang et al. that were inoculated with LAB without any additional stimulation, showing an increase in macrophage/monocyte and dendritic cell proportions in the blood, compared to a group that was inoculated with human rotavirus where an increase of the aforementioned cell count was observed in lymphoid tissue, with a relative decrease in the blood, in turn supporting the proposition that probiotic LAB might be responsible for the increase in macrophage/monocyte and dendritic cell proportion circulating in the bloodstream [44]. Similarly, studies involving other animal species and probiotic LAB strains showed an increase in the antigen-presenting cell population. An increase in dendritic cell and macrophage proportions has previously been reported, using *Lactobacillus plantarum* strain as a probiotic supplement in mice [45].

As has been outlined before, compounds in bovine colostrum are capable of up-regulating IL-10 production, which is responsible for the suppression of the inflammatory response, including down-regulation of macrophage cell proliferation, offering results contrary to the effect of probiotic LAB described above [40,46]. The case in which both probiotic LAB and bovine colostrum components can be considered anti-inflammatory is the evaluation of the macrophage cell proportion and activity in tissue, rather than in the bloodstream. A decrease in tissue macrophage/monocyte population can be associated with a relative increase in blood monocyte proportion. IL-10, produced by T regulatory cells, is mainly associated with macrophage-suppressing effect in tissue inflammation site, while suppression of monocyte activity in the blood is carried out in autoregulatory fashion by IL-10 produced by monocytes themselves [46]. In turn, the role of IL-10 in macrophage/monocyte changes associated with nutraceutical used in this study should be evaluated in order to explain the mechanism of their quantitative changes.

Different active compounds found in apples have previously been described to demonstrate anti-inflammatory and anti-allergic properties. However, only a limited number of studies that evaluate the effect of these compounds on macrophage/monocyte cell dynamics exist. Apples are particularly rich in flavonoids that are associated with a number of health benefits [47]. Flavonoids have previously been shown to prevent macrophage and T cell migration and reduce their numbers in the central nervous system in rats with experimentally induced allergic encephalitis, however, the mechanism of action is not well understood [48]. These findings indicate a similar effect of flavonoids to the one demonstrated by LAB and bovine colostrum, namely decrease of macrophage cells at tissue sites of inflammation and relatively elevated proportion of macrophage/monocyte cells in the blood. Similarly, Kanda et al. investigated the effect of apple polyphenol on the mast cell histamine release, indicating anti-inflammatory effects of crude apple polyphenol and apple condensed tannin (ACT) [19]. It is argued that histamine release suppression by ACT is achieved by inhibition of calcium influx, which in turn inhibits the activation of the signal transduction system preventing the release of intracellular secondary granule content. However, mast cells secret a number of other active substances, including pro-inflammation mediators, including CCL3 and TNFα, both of which play a role in macrophage chemotaxis and activation, respectively, using release via secretory granules [49]. Therefore, suppression of calcium influx by ACT might also be responsible for inhibiting the release of other pro-inflammatory mediators, including CCL3 and TNFα, in turn limiting the recruitment of macrophage/monocyte cells at inflammation sites. However, more studies involving animals with experimentally induced inflammatory conditions need to be carried out in order to investigate anti-inflammatory properties of a nutraceutical used in this study.

In all tested animals, *Bacteroidia* (phylum Bacteroidetes) and *Clostridia* (phylum Firmicutes) were the most prevalent bacterial classes found in feces, with prevalence rates ranging from 69.2% to 73.2%. These results are consistent with data reported by other authors, reporting Bacteroidetes and Firmicutes to be the most prevalent phyla in healthy piglet feces [50,51]. Microbial changes within Firmicutes 7 DPI included a decrease in the proportion of *Clostridia* and an increase in the proportion of bacilli, however, this shift was observed in both control and test animal groups. On the genus level, the difference between control and test groups was more pronounced, with a 2.7 times increase in *Lactobacillus* in the test group, compared to the control. This is consistent with the fact that one of the components of the nutraceutical used was *L. uvarum*. Lactobacilli are known to adjust easily to the intestinal environment, inhibit the growth of or kill pathogenic microorganisms in the gastrointestinal tract and improve the microbial balance of the gut, as well as maintaining intestinal barrier function [52].

At 14 DPI, an increase in the proportion of *Clostridia* was particularly visible in the test animal group, dominated by *C. cellulovorans*. This species is cellulolytic, capable of hydrolysis of lignocellulose, which results in the production of usable sugars. The shift towards cellulolytic bacteria in the test animal group can be associated with the nutraceutical supplement containing cellulose from DAP. It can be assumed that dietary components rich in cellulose, as is the case with the nutraceutical used in this study, might elevate the number of cellulolytic bacteria in pigs. Interestingly, an increase in *Barnesiella intestinihominis* was observed in the male control group. A limited amount of data is currently available on the importance of this species in pigs. *B. intestinihominis* has been associated with a role in cancer prevention of intestinal and extraintestinal origin [53], however, the shift of this species was short and was no longer observed at 28DPI, with no statistically significant difference between control and test group animals.

At 28DPI, the number of *Clostridia* remained higher in the test group compared to the control. In addition, a shift towards bacterial class Coriobacteria was observed in the test group. Among the Coriobacteria, two genera-*Collinsella* and *Olsenella*-were the most prevalent. Species of *Collinsella* are known for their ability to ferment a wide range of carbohydrates. The data from pigs reports a correlation between the number of *Collinsella* and the pre-weaned weight gain [54], which is consistent with our data (Table 1). Genus *Olsenella* consists of anaerobic LAB, however, they are not considered probiotic, requiring more investigation on their probiotic properties [55]. Overall, the total amount of probiotic bacteria was higher in the test animal group, indicating the positive effect of the nutraceutical on the microbiota of the gut. However, proportions of *Lactobacillus* and *Escherichia* were nonetheless higher in the control animal group (Figure 6). This relatively lower number of *Lactobacillus* in the test animal group could be explained by the relative increase in other lactic bacteria, such as *Olsenella* and *Bifidobacterium*. Moreover, proportions of *Lactobacillus* and *Escherichia* were overall relatively low, compared to other genera present in the gut.

## 5. Conclusions

Our study shows a positive effect of the nutraceutical formula used in this study on the changes of gut microbiota by an facilitating increase in probiotic bacteria strains and possible anti-inflammatory properties. Therefore, the combination of components used in this study could be used as a supplement in order to bolster the proportion of beneficial gut microorganisms, in addition to protection against conditions associated with inflammatory processes. However, the combination of ingredients used in the nutraceutical supplement has to be investigated further. In particular, test subjects with underlying inflammatory and immune system-related conditions have to be used in order to evaluate more accurately the effect of the nutraceutical supplement for its immunomodulatory properties. Moreover, hosts of other animal species have to be used in order to test the cross-species applicability of the supplement.

## 6. Patents

Multicomponent nutraceutical supplement used in the present study has been submitted to European Patent Office and registered under the title ‘Multifunctional Nutraceutical Composition Modeling the Gastrointestinal Microbiota and Immune System Response` (WIPO`s Digital Access Service access code C038).

## Figures and Tables

**Table 1:** Cholesterol, immunoglobulin and weight gain measurement comparisons between test and control groups at 28 DPI.

Parameter	Control Group	Test Group
Weight gain (kg)	10.71 ± 0.99	11.65 ± 1.30
IgA (g/L)	0.078 ± 0.06	0.11 ± 0.04
IgG (g/L) *	3.90 ± 0.48	4.68 ± 0.42
IgM (g/L)	0.57 ± 0.17	0.73 ± 0.25
Total cholesterol (mmol/l) *	1.89 ± 0.38	2.34 ± 0.34
HDL cholesterol (mmol/l)	0.68 ± 0.26	0.85 ± 0.11

* Significant results between the groups (*p* ≤ 0.05).

## Data Availability

All data generated or analysed during this study are included in this published article and supplementary material files.

## Supplementary Materials

The following are available online at https://www.mdpi.com/article/10.3390/foods10061313/s1, Table S1: Bacterial classes in female negative control group intestinal microbiota 28 days after supplementary feeding., Table S2: Bacterial classes in male negative control group intestinal microbiota 28 days after supplementary feeding.
